# The Role of Oxytocin in Cardiovascular Protection

**DOI:** 10.3389/fpsyg.2020.02139

**Published:** 2020-08-25

**Authors:** Marek Jankowski, Tom L. Broderick, Jolanta Gutkowska

**Affiliations:** ^1^Cardiovascular Biochemistry Laboratory, University of Montreal Hospital Centre, Montreal, QC, Canada; ^2^Department of Medicine, University of Montreal, Montreal, QC, Canada; ^3^Laboratory of Diabetes and Exercise Metabolism, Department of Physiology, College of Graduate Studies, Midwestern University, Glendale, AZ, United States

**Keywords:** oxytocin, oxytocin—therapeutic use, heart, atrial natriuretic peptide, cardiomyocyte

## Abstract

The beneficial effects of oxytocin on infarct size and functional recovery of the ischemic reperfused heart are well documented. The mechanisms for this cardioprotection are not well defined. Evidence indicates that oxytocin treatment improves cardiac work, reduces apoptosis and inflammation, and increases scar vascularization. Oxytocin-mediated cytoprotection involves the production of cGMP stimulated by local release of atrial natriuretic peptide and synthesis of nitric oxide. Treatment with oxytocin reduces the expression of proinflammatory cytokines and reduces immune cell infiltration. Oxytocin also stimulates differentiation stem cells to cardiomyocyte lineages as well as generation of endothelial and smooth muscle cells, promoting angiogenesis. The beneficial actions of oxytocin may include the increase in glucose uptake by cardiomyocytes, reduction in cardiomyocyte hypertrophy, decrease in oxidative stress, and mitochondrial protection of several cell types. In cardiac and cellular models of ischemia and reperfusion, acute administration of oxytocin at the onset of reperfusion enhances cardiomyocyte viability and function by activating Pi3K and Akt phosphorylation and downstream cellular signaling. Reperfusion injury salvage kinase and signal transducer and activator of transcription proteins cardioprotective pathways are involved. Oxytocin is cardioprotective by reducing the inflammatory response and improving cardiovascular and metabolic function. Because of its pleiotropic nature, this peptide demonstrates a clear potential for the treatment of cardiovascular pathologies. In this review, we discuss the possible cellular mechanisms of action of oxytocin involved in cardioprotection.

## Oxytocin and the Cardiovascular and Cardiorenal Systems

Our research in the last two decades on oxytocin (OT) in heart biology has generated a broad interest of the role of this neuropeptide on overall cardiometabolic and vascular functions. Our important findings highlighted the discovery of a specific OT system, including the presence of OT and the OT receptor (OTR) in the rodent and human heart (Gutkowska et al., 1997; Jankowski et al., 1998). The presence of OT and OTRs in cardiac chambers indicates autocrine and/or paracrine roles for this peptide. The effects of OT and its associated signaling pathways are mediated by OTRs, which are also present in large vessels (Jankowski et al., 1998, 2000) as well as in cardiac microvessels expressing the CD31 marker and co-localizing with endothelial nitric oxide (NO) synthase (eNOS) (Thibonnier et al., 1999; Jankowski et al., 2010b). The adult rat heart expresses the OTR in all chambers, but it is most abundantly found in the endothelium and cardiomyocytes (CMC) (Jankowski et al., 2004b; Wang et al., 2003). OT exerts its functions by binding to OTRs in cardiac cells or indirectly in the vasculature to regulate function, such as decreasing the left ventricular (LV) preload and the inotropic state (Jankowski et al., 1998). Vascular OT is involved in control of vascular tone and blood flow, regrowth, and remodeling (Thibonnier et al., 1999; Cattaneo et al., 2009); and depending on the vascular bed, OT induces both vasoconstriction and vasodilation (Japundzic-Zigon, 2013). As a result, systemic administration of OT has significant effects on vascular tone, blood flow and pressure, and cardiovascular regulation (Gutkowska et al., 2014) *via* neural (Uvnäs-Moberg et al., 2019) and renal effects (Jankowski et al., 2019). Conversely, OT or OTR knockout mice do not appear to demonstrate deficiencies in cardiac function (Jankowski et al., 2016). However, cardiovascular function is impaired in obese aged mouse models deficient in OTR function (Takayanagi et al., 2008). In fact, differences were noted in basal blood pressure, baroreflex function, and autonomic function in OT-deficient mice, supporting the role of OT system in cardiovascular function (Michelini et al., 2003).

**FIGURE 1:**
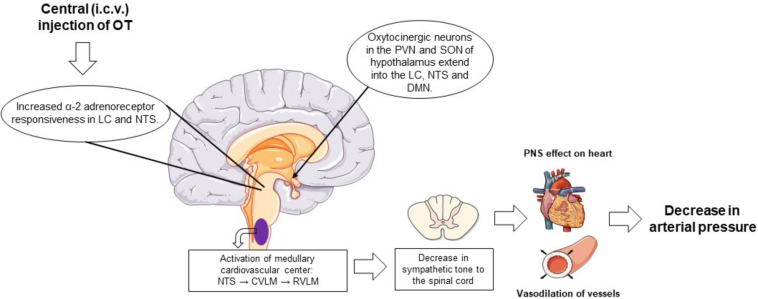
Schematic representation of the centrally mediated actions of oxytocin treatment on blood pressure regulation. Intracerebrovascular injections of OT increase α-2 adrenoreceptor responsiveness in the LC and NTS. Within the NTS, impulses reach the medullary cardiovascular center of the medulla oblongata where the baroreceptor afferents are activated to variations in arterial pressure. When arterial pressure is elevated or in with injections of OT, inhibitory neurons in the CVLM that extend to the RVLM, which regulates sympathetic nervous tone from the spinal cord to peripheral organs, are activated. Activation of this pathway suppresses peripheral sympathetic outflow to the heart and peripheral resistance vessels, leading to bradycardia and vasodilation of these vessels, respectively, and a decrease in arterial pressure. In addition, intracerebrovascular administered OT activates the oxytocinergic neurons and stimulates the synthesis and release of OT from the posterior pituitary. Plasma OT binds to the OTRs in cardiac tissue to induce a bradycardia and the release of ANP. OT also binds to the OTRs present in the vasculature, causing vasodilation. ANP, atrial natriuretic peptide; CVLM, caudal ventrolateral medulla; DMN, dorsal motor nucleus of the vagus nerve; LC, locus coeruleus; NTS, nucleus tractus solitarius; OT, oxytocin; OTR, oxytocin receptor; PVN, paraventricular nuclei; RVLM, rostral ventrolateral medulla; SON, supraoptic nuclei.

**FIGURE 2:**
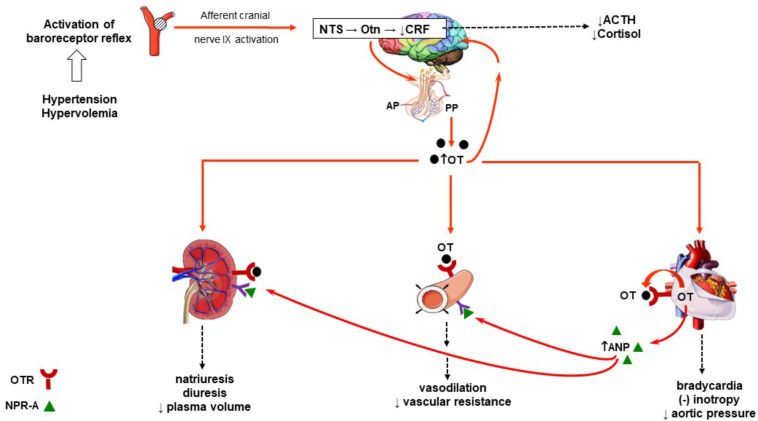
Schematic diagram of the role of OT in the regulation arterial blood pressure. Activation of the baroreceptor (renal, aortic, and carotid) reflex to an increase in blood volume expansion or hypertension and subsequent integration of the afferent signals within the NTS activates oxytocinergic neurons. This induces the synthesis of OT from the PVN and SON of the hypothalamus and release into the plasma. In plasma, OT can bind to OTRs found in the heart, kidney, and vasculature. The heart is also a source of OT where it can also bind to its receptors. Activation of the OTR in the heart induces bradycardia and a decrease in inotropy as well as the release of ANP and NO. Release of cardiac ANP induces vasodilation of peripheral arterioles after binding to the NPR-A. The effect of OT on peripheral vasodilation is a NO-dependent vasodilation effect. ANP also binds to NPR-A receptors in the renal vascular. Physiological concentrations of OT and ANP induce arteriolar dilation, leading to diuresis, natriuresis, kaliuresis, and a decrease in plasma volume. The net effect is a decrease in arterial pressure. High levels of OT in either plasma or centrally produced from the oxytocinergic system are known to suppress the hypothalamus–pituitary–adrenal axis. This decreases CRF release from the hypothalamus and decreases production of ACTH and cortisol. ACTH, adrenocorticotropic hormone; ANP, atrial natriuretic peptide; AP, anterior pituitary; CRF, corticotropin-releasing hormone; NO, nitric oxide; NPR-A, natriuretic peptide receptor, type A; NTS, nucleus tractus solitarius; OT, oxytocin; OTn, oxytocinergic neurons; OTR, oxytocin receptor; PP, posterior pituitary; PVN, paraventricular nuclei; SON, supraoptic nuclei.

The role of OT in the regulation of blood pressure is well documented (Petersson, 2002; Gutkowska and Jankowski, 2011; Gutkowska et al., 2014; Buemann and Uvnäs-Moberg, 2020). OT regulates arterial blood pressure by acting through both central and peripheral mechanisms. Immediate and rapid effects on blood pressure occur with direct intracerebrovascular injections of OT (Figure 1). The decrease in arterial pressure and bradycardia by central application of OT is associated with enhanced α-2 responsiveness in the locus coeruleus, nucleus tractus solitarius, and dorsal motor nucleus of the vagal nerve (Higa et al., 2002; Petersson, 2002; Buemann and Uvnäs-Moberg, 2020). Subcutaneous injections of OT can also decrease blood pressure by acting peripherally on the cardiovascular and renal systems. By acting through these systems, OT decreases arterial blood pressure by modulating the autonomic nervous system, resulting in a reduction in heart rate and contractility. In addition, OT decreases vascular resistance of peripheral blood vessels and increases renal blood flow, producing a natriuretic effect and a decrease in blood volume. Atrial natriuretic peptide (ANP) release from atrial CMC mediated by a paracrine effect of OT also favorably decreases arterial pressure. This indirectly inhibits the synthesis of renin and aldosterone and induces diuresis (Figure 2). Interestingly, with repetitive subcutaneous injections of OT, this peptide crosses the blood–brain barrier (BBB) and decreases arterial pressure by acting on central mechanisms.

## Oxytocin Potential in Heart Regeneration

Enhanced OT synthesis and OTR expression have been reported in the developing heart at day 21 of gestation and early postnatal days (Jankowski et al., 2004a). At this stage, cardiac cells intensively proliferate *in vitro* and contain numerous cardiac progenitor cells (CPCs), which can contribute to cardiac reparative processes (Bollini et al., 2011). Several studies have proposed a role for OT in cell proliferation (Emmert et al., 2012; Noiseux et al., 2012), and therefore, we proposed that OT is involved in differentiation stem cells into cardiac lineages. For differentiation, the well-established mouse embryonal carcinoma P19 cell line for early heart differentiation was employed (van der Heyden and Defize, 2003). The efficient differentiation of P19 cells depends on the prior formation of non-adhering cell aggregates, which can differentiate into all three germ layers. Treatment aggregates with 10^–7^ M of OT resulted in generation of beating cell colonies, which were disclosed as CMC using histological, molecular, and electrophysiological markers (Paquin et al., 2002). Next, we revealed that activation of NO plays an important role in OT-mediated CMC differentiation (Danalache et al., 2007) and that OT stimulates differentiation of endothelial and smooth muscle cells (Cattaneo et al., 2009), promoting angiogenesis. A number of reports revealed that cardiomyogenesis occurs in different stem cell lines, including somatic stem cells (Sca-1) (Matsuura et al., 2014) isolated from adult mouse and rat cardiac stem cell progenitors identified as side population (Oyama et al., 2007). These and other findings suggested that OT serves as a naturally occurring cardiomorphogen. This is supported by observation that OT and its extended form, which contain the amino acids glycine, lysine, and arginine (OT-GLA), are abundant in the mouse fetal heart (Danalache et al., 2010). In contrast to the OT system in the hypothalamus, both OT and OTR are elevated in postnatal CMC and then decrease to low levels in adults (Jankowski et al., 2004a). Interestingly, the well-recognized and major cardiomyogen retinoic acid increases the expression of OT in the fetal heart (Jankowski et al., 2004a). Correspondingly, the generation of functional CMC from stem cells by OT (Paquin et al., 2002; Gassanov et al., 2008; Matsuura et al., 2014) has been proposed for cardiac cell therapy (Kim et al., 2010; Noiseux et al., 2012).

## Oxytocin in Cardiovascular Pathologies

The importance of the cardiovascular actions of OT was also acknowledged in recently published reviews in relation to atherosclerosis (Wang et al., 2019) and in the prevention and treatment of cardiovascular pathologies (Reiss et al., 2019; Buemann and Uvnäs-Moberg, 2020). Taken together, many of these observations indicate that OT plays a critical role on cardiovascular function. Since the first investigation describing the cardiac OT system, it was clearly established that in addition to the direct action of OT on cardiovascular regulation, the effects of OT are also mediated by the release of ANP (Gutkowska et al., 1997). OT stimulates the release of ANP from CMC through endocrine and paracrine pathways, which in turn regulates blood pressure via both renal excretion mechanisms and peripheral vasodilation. OT also regulates coronary perfusion pressure by inducing vasodilation of coronary resistance arteries (Gutkowska et al., 1997). ANP is also involved in many beneficial actions in the heart, such as reduction of extracellular fluid, improvement in cardiac ejection fraction, and inhibition of both CMC hypertrophy and fibrosis in heart failure (Gutkowska et al., 2014). ANP and brain natriuretic peptide (BNP) are potent diuretic, natriuretic, and vasorelaxant hormones synthetized in the heart, brain, and various peripheral tissues (Gutkowska and Nemer, 1989). Secondly, NO, a key regulator of cardiac and vascular function, is involved in numerous actions in the heart and vascular beds by OT (Gutkowska et al., 2014). A number of social and reproductive behaviors such as breastfeeding, cutaneous contact between a mother and her infant, and sexual activity either alone with or others are linked to OT sensory stimulation (Buemann and Uvnäs-Moberg, 2020; Szczepanska-Sadowska et al., 2020). In addition, OT release is associated with reduced anxiety, depression, pain, inflammation, a reduction in the risk of cardiovascular disease and diabetes (Uvnäs-Moberg et al., 2014). On the other hand, negative social and behavioral factors can contribute to lowering the levels of OT in plasma (Tsai et al., 2019). This can result in decreased expression of OTR (Nation et al., 2010) and on a chronic basis leading to the development of cardiovascular abnormalities.

Patients with diabetes are at increased risk of developing cardiovascular diseases. In addition, the severity of cardiac ischemic events is greater in patients with type 2 diabetes (T2DM). Epidemiological studies have shown that death rates from cardiovascular diseases are increased in the patients with T2DM. This higher mortality rate is explained, in part, the downregulation of the pro-survival kinase pathways, disturbances in the mitochondrial permeability transition pore (mPTP), dysfunctional mitochondrial adenosine triphosphate (ATP)-sensitive potassium channel (mKATP) channels, and increased calcineurin activity, all of which are linked to loss of conditioning (Penna et al., 2020b). The role of OT on glucose uptake, pancreatic function, and regulation of body weight balance suggests the involvement of OT in the pathophysiology of diabetes (Elabd and Sabry, 2015). These effects were observed not only in animal models but also in human clinical studies (Zhang et al., 2013; Klement et al., 2017). However, few studies to date have examined the cardioprotective effects of OT in animal models of diabetes. This is explained by the lack of available relevant animal models of disease that precisely mimic the human pathology associated with diabetes and the difficulty in adapting during the events of ischemia and reperfusion (IR) that occur in the human heart (Russo et al., 2017; Penna et al., 2020b). Kim Y.S. et al. (2013) obtained indirect proof that OT might provide cardiac protection against diabetes by demonstrating that the lack of therapeutic action of mesenchymal cells isolated from diabetic rat was ameliorated after cell treatment in medium supplemented with OT. The db/db mouse model is a commonly used model of insulin resistance, system inflammation, and obesity for the study of T2DM. This model also exhibits many of the metabolic and cardiovascular function changes recognized with human T2DM. These mice also display aspects of psychosis and depression-like behaviors as seen in some psychiatric disorders (Ernst et al., 2013). The db/db mouse displays augmented cardiac apoptosis, CMC enlargement, collagen accumulation, and a significant downregulation in cardioprotective genes related to the OT–NP–NO system. Our studies have demonstrated that the expression of OT, OTR, ANP, BNP, and eNOS is reduced by more than 50% in hearts from db/db mice compared with lean wild-type control mice (Gutkowska et al., 2009). Our new evidence demonstrated that OT treatment of young db/db mice prevented the onset cardiomyopathy from occurring (Plante et al., 2015). Similarly, inhibition of diabetic cardiomyopathy in this model of diabetes has been demonstrated by treatment with BNP, which further provides evidence that the beneficial effects of OT on heart function are mediated, at least in part, by natriuretic peptides (Plante et al., 2014). More data regarding the effects of OT in diabetic models of cardiovascular pathology were published in our recent review (Jankowski et al., 2016). In the following section, we discuss evidence supporting the role and interactions between OTR and the factors involved in the protection of cardiac cells as well as how these can potentially regulate mitochondrial processes.

## Protective Role of the Oxytocin Receptor in the Heart

Coronary heart disease (CHD) in the form of acute myocardial infarction (MI) and acute coronary syndromes accounts for nearly one-third of heart disease-related deaths and disability in developed countries. Timely reperfusion of the coronary arteries after acute MI is critical for the restoration of blood flow. A reduction in infarct size can also be accomplished by subjecting the heart to repeated short periods of ischemia followed by IR (Penna et al., 2015). Rapid restoration of blood flow and oxygen delivery during reperfusion after ischemia paradoxically induces cell dysfunction and death but is essential to protect ischemic tissues and restore function. However, conditioning strategies are known to reduce damage to the reperfusion heart. In the ischemic preconditioning (IPC) setting, this is accomplished by inducing brief non-lethal periods of IR before a longer and sustained phase of IR to the heart or to peripheral organs [remote ischemic preconditioning (RIPC)]. Postconditioning (IPostC) refers to this process performed at the onset of reperfusion. Preclinical studies on experimental animal models have identified numerous molecular pathways potentially involved in cell death and accessible for therapeutic intervention (Ruiz-Meana et al., 2019). However, application of these cardioprotective approaches into the relevant clinical realm has been disappointing, and many potential reasons for this have been proposed (Kleinbongard et al., 2019). First, patients recruited into these trials are generally at an advanced age and display several confounding features like hyperlipidemia, hypertension, obesity, and diabetes; these conditions are difficult to reproduce in animal models used for research purposes (Russo et al., 2017; Ruiz-Meana et al., 2019; Femmino et al., 2020; Penna et al., 2020b). Second, since MI is multifactorial, CMC death occurs *via* multiple mechanisms and pathways, affecting platelets, fibroblasts, endothelial cell, smooth muscle cells, and immune cells. In this regard, optimal cardioprotection likely requires the application of several combinations of additive or synergistic multitarget therapies (Davidson et al., 2019). In this context, the pleiotropic nature of OT is an attractive hormone that could provide added benefits in the treatment in cardiovascular pathologies (Gutkowska and Jankowski, 2012). There is growing recognition of the anti-ischemic actions of OT in experimental models of IR injury (see Table 1). In rat and rabbit models of ischemic heart disease, OT enhances recovery of LV function by reducing ischemic and reperfusion damage (Kobayashi et al., 2009; Jankowski et al., 2010a; Ondrejcakova et al., 2012; Alizadeh and Mirzabeglo, 2013) and infarct size (Kobayashi et al., 2009; Ondrejcakova et al., 2009; Alizadeh et al., 2010; Jankowski et al., 2010a). Downregulation of the OT–OTR system induced by experimental MI can be reversed with OT administered either before the onset of ischemia or 7 days after the ischemic injury (Jankowski et al., 2010b). In addition, OT treatment improved cardiac work, increased scar vascularization, and reduced apoptosis. This anti-apoptotic effect of OT involved the OTR based on our experiments using H9c2 cells transfected with siRNA sequences complementary to OTR mRNA. In cells expressing reduced OTR content, treatment with OT enhanced apoptotic effect of IR, whereas in control cells treated with scrambled siRNA, OT evoked significant protection (Gonzalez-Reyes et al., 2015). These effects are consistent with observation that in the early period after MI in the rat, mRNA expression of OTR is significantly downregulated and then gradually increases above the normal levels following OT treatment (Jankowski et al., 2010b).

**TABLE 1:** The effects of oxytocin in various cardioprotective models.

Function	Target	Mechanism	References
Negative inotropy and chronotropy.	Isolated dog right atria. Isolated, perfused rat heart.	Activation intrinsic cardiac cholinergic neurons and NO. ANP-releasing action of OT.	Favaretto et al., 1997; Gutkowska et al., 1997; Mukaddam-Daher et al., 2001; Costa-E-Sousa et al., 2005
Inhibition of CMC hypertrophy.	Rodent CMC in culture stimulated by endothelin-1.	cGMP, calcium-calmodulin kinase kinase and AMP-activated protein kinase pathway, Akt phosphorylation and NFAT.	Menaouar et al., 2014
Glucose uptake in CMC.	Primary cultures of neonatal rat CMC.	NO and PI3K, Ca-CAMKK and AMPK pathways.	Florian et al., 2010
OT preconditioning in ischemia and reperfusion.	Anesthetized rats. Isolated perfused rat heart. Anesthetized rabbits. Anesthetized rats.	Cardioprotection was blocked by ANP inhibitor, anantin. Cardioprotection was reduced in the absence of negative inotropy and chronotropy. Cardioprotection was blocked by OTR, mitochondrial K_ATP_ channels and NO. Cardioprotection was blocked by inhibitors of the mitoKATP channel, and an mPTP opener.	Ondrejcakova et al., 2009; Alizadeh et al., 2012; Das and Sarkar, 2012; Houshmand et al., 2015
OT postconditioning in ischemia reperfusion.	Infarcted rabbits. Isolated perfused rat heart.	Infarct reduction, improvement cardiac functional parameters, expression of cardioprotective genes. OT dose-dependent reduction in infarct size. Cardioprotection reduced by PI3K/Akt, ERK1/2 inhibitors and Atosiban. Involvement SAFE pathway in cardioprotection.	Kobayashi et al., 2009; Anvari et al., 2012; Polshekan et al., 2016, 2019
OT treatment in simulated ischemia reperfusion.	H9C2 cardiomyoblasts.	OT treatment most effective in early reperfusion. Activation of ERK1/2, Pi3K/Akt, and eNOS with rapid NO release. OTR trafficking to mitochondria in signalosomes.	Gonzalez-Reyes et al., 2015
Chronic OT treatment.	Infarcted rats. Ischemia and reperfusion of isolated, perfused rat heart. Infarcted porcine.	Reduction of inflammation and apoptosis in infarcted and remote myocardium; improved heart function. Functional and structural cardioprotection, activation p38-MAPK, ANP, HSP27 and Akt kinase pathways. No significant effects of treatment on structural and functional cardiac parameters.	Authier et al., 2010; Jankowski et al., 2010b; Ondrejcakova et al., 2012
CMC co-culture with OT preconditioned rat MC. Treatment with OT conditioned MC. Treatment with OT conditioned MC. isolated from STZ-induced diabetic rats.	Rat newborn CMC exposed to ischemia reperfusion. Ischemia/reperfusion in rats. Ischemia/reperfusion in rat.	Inhibition of apoptosis with secretion of factors associated with angiogenesis and anti-cardiac remodeling. Improvement of structural and functional cardiac parameters. Reduction in angiogenic capacity and therapeutic potential of diabetic MC were restored by OT treatment.	Kim Y.S. et al., 2012; Noiseux et al., 2012; Kim Y.S. et al., 2012
OT treatment of rats before heterotopic heart transplantation.	Rat hearts exposed to ischemia reperfusion during transplantation.	Downregulation of the inflammatory response, ROS and neutrophil-dependent apoptosis.	Al-Amran and Shahkolahi, 2013

*AMPK, AMP-activated protein kinase; ANP, atrial natriuretic peptide; Ca-CAMKK, calcium-calmodulin-dependent protein kinase; CMC, cardiomyocytes; eNOS, endothelial nitric oxide synthase; HSP, heat shock protein; MC, mesenchymal cells; mPTP, mitochondrial permeability transition pore; NFAT, nuclear factor of activated T-cells; NO, nitric oxide; OT, oxytocin; OTR, oxytocin receptor; ROS, reactive oxygen species; SAFE, survivor activating factor enhancement; STZ, streptozotocin*.

In addition to the well-documented roles of OT in classic reproduction functions, evidence suggests that activation of OT and AVP-mediated signaling may benefit cardiovascular function in pregnancy (Szczepanska-Sadowska et al., 2020). It is suggested that stimulation of the OT and AVP receptors in the cardiovascular system protects cardiovascular function in the mother and fetus. However, there are few reports that inappropriate action of OT after bolus injection at high OT concentration may be detrimental to the cardiovascular system and increase the risk of heart failure in the mother and offspring (Svanstrom et al., 2008). In contrast, we have demonstrated the presence of fibrotic deposits, CMC hypertrophy, capillary rarefaction in heart, and increased expression of cardiac pathology markers in the pregnant rat following placental ischemia (Gutkowska et al., 2011). Some of these detrimental consequences were reversed with etanercept treatment, a soluble receptor of TNF-α, which activated eNOS and enhanced OTR identified in cardiac microvessels (Gutkowska et al., 2011). Also, the activation of the OTR using the estrogenic stimulus of genistein in ovariectomized rats was potentially associated with improvement of aortic structure (Wang et al., 2003) and cardiac functional parameters (Jankowski et al., 2010b). In examining the role of this system as being either beneficial or detrimental, it is important to consider the fine balance that exists between cardiac OTR versus the OT levels produced locally and the concentration of OT present in the circulation. Moreover, the published data raise the important question of how naturally occurring variations in the cardiac OT system can influence pathology and physiological processing of OT in the heart. Also of importance in this cardiac-specific overexpression of OTR model is the role of the arginine-vasopressin system (AVP; hormone and V_1_/V_2_ receptors) and its potential involvement on cardiac function (Gutkowska et al., 2014). Although OT and AVP have a higher affinities for their own receptors, cross-talk between these hormones with OT and AVP receptors is a likelihood when these peptides are administered in high concentrations (Song and Albers, 2018). For example, depending on the concentration and route of administration, OT induces diuretic and antidiuretic responses in rat kidney (Balment et al., 1980). With the use of a physiological concentration, diuresis and natriuresis are stimulated in rodents (Jankowski et al., 2019) by mechanisms that involve the release of ANP by the heart and activation of OTR in kidney to produce NO (Haanwinckel et al., 1995; Soares et al., 1999). In the presence of a pharmacological concentration of OT (Ecelbarger et al., 2001; Moeller and Fenton, 2012) or chronic infusion, an antidiuretic effect is observed mediated by the V_2_ receptor. In addition, the OTR and similar receptors (i.e., rhodopsin-type Class 1) of the G-protein coupled receptors is regulated by changes in receptor expression and resistance and local OT concentrations exposed to the OTR (Gimpl and Fahrenholz, 2001). Therefore, from a pharmacotherapy perspective, OT analogs should be developed to offer both safe and efficacious treatment for the treatment of cardiovascular diseases (Buemann and Uvnäs-Moberg, 2020). In fact, postconditioning with OT decreased infarct size in a U-shaped dose-dependent manner with a maximum cardioprotective effectiveness achieved with 10^–11^ M (Anvari et al., 2012), a concentration that nearly matches the physiological level of OT measured in rat plasma (Leng and Sabatier, 2016). Therefore, OT concentration and duration of treatment, density and affinity of specific receptors, the presence of accompanied AVP receptors, and other factors decide about beneficial or deleterious effects on the cell, organ, and whole body.

## Oxytocin and Inflammation

Nervous and immune systems display similar functions such as monitoring and rapidly responding to imbalance in the cardiovascular homeostasis (Carnevale and Lembo, 2020). The innate immune response in peripheral tissues is regulated by the neuro-immune circuit in central nervous system [reviewed in Irwin and Cole (2011)]. The innate immune system also in reverse, by production of cytokines, can regulate the function of the central nervous system, and this has effects on behavior (Kenney and Ganta, 2014). Accumulating evidences indicate that the hypothalamo-neurohypophysial system is involved in neuroendocrine–immune network, wherein the OT-producing system plays an important function (Li et al., 2016). Petersson et al. (1998) demonstrated that low dose of OT given intracerebroventricularly mediated mechanisms in the brain regulating survival of dorsal musculocutaneous flaps was improved in rats. As recently discussed, the anti-inflammatory effects of OT may be delivered from the hypothalamus/posterior pituitary gland both by humoral routes and by the autonomous nervous system (Buemann and Uvnäs-Moberg, 2020). Several pathophysiological events (as illustrated on Figure 2) lead to activation neural circuit and cardiac release of ANP, also known as an important factor in innate immune function as well as in the adaptive immune response (Vollmar, 2005). It has been reported that inhibition of neurohypophysial hormones secretion by hypophysectomy and neurointermediate pituitary lobectomy in rat blocked humoral and cellular immune responses in rats (Quintanar-Stephano et al., 2004; Campos-Rodriguez et al., 2006). An accelerated healing of skin wound in mice and humans was obtained by treatment with bacteria, *Lactobacillus reuteri* (Poutahidis et al., 2013). OT was essential for this wound-healing effect, because both OT knockout and vagotomy abolished skin wound closure (Poutahidis et al., 2013). Since the most of neurons in the vagal nerve are afferent (Forsythe et al., 2014), therefore, it is possible that neural signals were transmitted from the intestine to the CNS and then stimulated OT anti-inflammatory response at periphery. The vagus nerve provides innervation to a wide variety of tissues, including the heart, and its activation is stimulated by numerous factors like mechanosensitive receptors in the cardiovascular systems (Carnevale and Lembo, 2020).

Cardiovascular pathology is characterized by oxidative stress and inflammation and release of inflammatory cytokines (Ong et al., 2018; Zuurbier et al., 2019). Dying CMC cause an inflammatory response from increased production of damage-associated molecular patterns (DAMPs), reactive oxygen species (ROS), and complement. The release of proinflammatory cytokines mediates the accumulation of neutrophils, monocytes, macrophages, B lymphocytes, and CD8+ T cells into the infarct zone (Ong et al., 2018; Zuurbier et al., 2019). DAMPs serve as ligands for pattern recognition receptors (PRRs), including Toll-like receptors (TLRs) and nucleotide-binding oligomerization domain-like receptor family of cytosolic protein (NLRP3) inflammasomes. As recently discussed, the anti-inflammatory effects of OT have been supported under local and systemic levels (Buemann and Uvnäs-Moberg, 2020). There are recent reviews documenting that OT alleviates these immunoinflammatory abnormalities (Li et al., 2016; Bordt et al., 2019) with three referring to the actions of OT in the cardiovascular system (Reiss et al., 2019; Wang et al., 2019; Szczepanska-Sadowska et al., 2020). We have observed that following MI, rats preconditioned with OT displayed improvement of cardiac contractile function. This beneficial effect on LV function was associated with reduced fibrosis and a diminished inflammatory response marked by decreases in neutrophils, macrophages, and T lymphocytes. In these hearts, OT also decreased the expression tumor necrosis factor α (TNF-α), interleukin (IL)-1β, and IL-6 (Jankowski et al., 2010b). In the absence of OT, these changes can affect function of the infarcted heart. Both IL-1β and TNF-α may reduce ATP production by inducing the production of ROS, thereby inhibiting oxygen use by the heart (Ebermann et al., 2009). OT treatment can restore the deficiency in ATP production by stimulating (Jankowski et al., 2010b) glucose uptake in CMC (Florian et al., 2010). The improvement of cardiac contractility and prevention of heart failure in response to OT treatment may be related to lowered TNF-α expression in the infarcted myocardium (Schumacher and Naga Prasad, 2018). In addition, infusions of OT stimulate the expression of transforming growth factor (TGF)-β, resulting in an improvement of LV function, lowered apoptosis, and enhanced cell proliferation (Frantz et al., 2008). OT can also stimulate gene expression of the anti-inflammatory cytokine IL-10 in LV scar tissue (Jankowski et al., 2010b). Because the mRNA from the infarct zone is derived mainly from cells invading the infarcted zone, the presented changes in cytokine mRNA may reflect the expression of local inflammatory cells. Indeed, Szeto et al. (2008) reported that within lipopolysaccharide (LPS)-stimulated human THP-1 macrophages, OT inhibits the secretion of the proinflammatory cytokine IL-6. A further study from this group indicated that human THP-1 cells, mouse macrophage cell lines, and primary human monocyte-derived macrophages treated with LPS produced a 10- to 250-fold upregulation of OTR mRNA with treatment with OT decreasing LPS-induced production of IL-6 (Szeto et al., 2017). In contrast to results reported using microglial cells, where OTR upregulation in response to LPS appears to be dependent on nuclear factor kappa-light-chain-enhancer of activated B cells (NF-κB) (Yuan et al., 2016; Inoue et al., 2019), the LPS-stimulated increase in OTR in macrophages was not blocked by inhibition of NF-κB but rather from inhibition of the ERK in the MAPK pathway by preventing phosphorylation of p38 (Szeto et al., 2017). The investigations on microglial cell models revealed several new observations, which potentially can be translated to the studies of OT in the cardiovascular system. Inoue et al. (2019) demonstrated that LPS treatment of microglia induced a stress reaction of the endoplasmic reticulum (ER). The stressed ER stimulates an unfolded protein response counteracting the accumulation of unfolded or misfolded proteins accomplished by activation of transcriptional or translational pathways to maintain cellular homeostasis (Wang et al., 2018; Bordt et al., 2019). An important mediator in this response is role of eukaryotic initiation factor 2α (eIF2α). In LPS-treated microglial cells, OT reduced the phosphorylation levels of eIF-2 α and subsequently suppressed signaling pathways related to the production of TNF-α and interleukins (IL-6 and IL-1β), as well as caspase-1 and caspase-11, markers of NLRP3 inflammasome (Inoue et al., 2019). In a study by Klein et al. (2016) using Caco2BB gut cells, OT treatment in combination with LPS significantly enhanced phosphor-IF2α levels as compared with only LPS, suggesting a protective role of OT in reducing protein translation to LPS treatment. Finally, recent results indicate that OT significantly reduced LPS-induced injury and prevented levels of IL-1β, IL-18, and IL-6 from increasing. Interestingly, OT also inhibited LPS-induced TLR4 expression and NLR family pyrin domain containing NLRP3 inflammasome activation (An et al., 2019).

The inflammasome is a multimolecular complex in the cell that is capable of detecting stresses and inducing inflammatory response. The inflammasome produces and releases active cytokines (primarily IL-1β), which mediate the acute phase of an inflammatory response as seen in fever. The involvement of the NLRP3 inflammasome in conditions such as impaired glucose tolerance and peripheral inflammation from obesity has been reported, and evidence suggests that the inflammasome may contribute to the development of vascular and ventricular dysfunction (Sokolova et al., 2019; Femmino et al., 2020). The most widely characterized inflammasome sensor in the heart is NACHT, LRR, and PYD domain-containing NLRP3, which is activated in response to sterile stimuli like cell debris during acute MI (Toldo and Abbate, 2018). Within the healthy heart, NLRP3 is slightly expressed and increases after 1–3 h of reperfusion in experimental IR in several animal models. Activation of the NLRP3 inflammasome stimulates additional myocardial damage by promoting IL-1β release and through induction of inflammatory cell death (Toldo et al., 2018). In diabetes, hyperglycemia and elevated free fatty acids promote gluco- and lipotoxicity that stimulate oxidative and ER stress, which in turn causes an inflammatory response by the NLRP3 inflammasome and related proinflammatory interleukins. Therefore, in diabetic subjects, NLRP3 expression may be elevated before an ischemic insult and leads to enhanced injury during an acute MI. Inhibiting NLRP3 inflammasome function early during reperfusion following acute MI is beneficial on recovery of LV function by reducing infarct size (Toldo and Abbate, 2018; Toldo et al., 2018). OT can play a significant role in this process.

## Molecular Signaling of Oxytocin in the Heart

In addition, OT maintains the viability and morphology of CMC in the injured heart. To explain this effect, several mechanisms of action of OT in cardiac cells have been proposed. These mechanisms include decreased apoptosis, hypertrophy, and fibrosis in CMC as well as an increase in glucose uptake and oxidation. Benefits of OT also occur as a result of increased cell proliferation and differentiation of cardiac stem cells (Gutkowska and Jankowski, 2011). We have reported that exogenous OT administration regulates the intrinsic cardiac conduction system, producing a negative chronotropic effect (Jankowski et al., 1998). OT induces a transient negative inotropic and chronotropic effect in isolated perfused dog right atria by increasing NO production and acetylcholine release on cardiac parasympathetic postganglionic neurons (Mukaddam-Daher et al., 2001). Decreasing chronotropicity of the heart reduces oxygen consumption and improves both coronary blood flow and subendocardial blood flow resulting in an increased contractile function (Ondrejcakova et al., 2009). The importance of these actions of OT on the intrinsic neural system in the heart was demonstrated by Jovanovic et al. (2019) where the uptake of norepinephrine and expression of cardio-inhibitory receptors were stimulated in the heart of chronically socially isolated animals treated with OT. This could clearly protect the cardiovascular system under conditions of stress when the activity of both the sympathetic and sympathoadrenal systems is activated.

**FIGURE 3:**
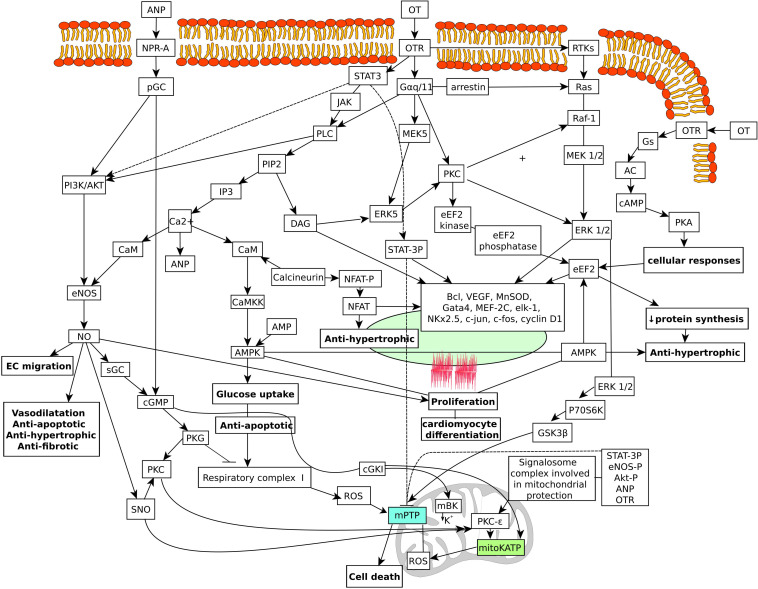
Schematic diagram of hypothetical OT signaling pathways targeting the nucleus and mitochondria in the cardiac cells. Multiple mechanisms stimulated by the OTR can contribute to the beneficial effects in cardiac cells. OTR activates GTP-binding proteins of the G alpha q/11 class, which stimulate phospholipase C activity, IP3 generation, and release of calcium from intracellular stores. Changes in calcium stimulate ANP release from cardiac atrial deposits and subsequent activation of particulate guanylyl cyclase and cGMP production. Activation of PI3K signaling pathway is also involved in cardioprotection. This results in phosphorylation and activation of AKT and NOS activation and NO production. NO stimulates soluble guanylyl cyclase also producing cGMP. cGMP has been linked to activate the mitochondrial mKATP channel. Activation of the PI3K/ERK1/2 pathways also leads to phosphorylation and inactivation of GSK. Inhibition of GSK has also been reported to inhibit the mPTP. Activation of the mKATP channels stimulates the inhibitory phosphorylation of GSK-3b and inhibits ROS production. NO also induces protein *S*-nitrosylation (SNO). SNO of the L-type calcium channel reduces calcium entry and the amount of calcium that enters the mitochondria. This inhibits the effect of calcium on the activation of the mPTP. SNO effects on complex I results in lowered production of ROS. In effect, these beneficial signaling pathways cause reduction in calcium and ROS triggers for mPTP. In addition, cGMP stimulates cGKIα, which interacts with mitochondrial large conductance calcium-activated potassium channel, stimulating potassium influx in mitochondria. This effect may lead closure of the mPTP and cardioprotection. ANP, atrial natriuretic peptide; NO, nitric oxide; OT, oxytocin; OTR, oxytocin receptor; ROS, reactive oxygen species. AC, adenylate cyclase; AMPK, AMP-activated protein kinase; AKT, protein kinase B; ANP, atrial natriuretic peptide; CaM, calmodulin; CaMKK, Ca^2+^ calmodulin-dependent protein kinase kinase; CaMK, Ca^2+^/calmodulin-dependent protein kinase; cGKI, cGMP-dependent protein kinase type Iα; cGMP, cyclic guanosine monophosphate (DAG) diacyl glycerol; (EC) endothelial cells; eEF2, elongation factor 2; ERK, extracellular signal-regulated kinases; GATA4, GATA binding protein 4; GSK3β, glycogen synthase kinase 3 beta; IP3, inositol 1,4,5-trisphosphate; JAK, Janus kinase; mBK, mitochondrial BKCa channel; MEK5, mitogen-activated protein kinase kinase 5; mKATP, mitochondrial K_ATP_ channels; MnSOD, manganese superoxide dismutase; NFAT, nuclear factor of activated T-cells; NOS, NO synthase; NPR-A, natriuretic peptide receptor A; pGC, particulate guanylyl cyclase; PIP2, phosphatidylinositol 4,5-bisphosphate; PKA, protein kinase A; PKC, protein kinase C; PKG, protein kinase G; PLC, phospholipase C; PI3K, phosphatidyl-3 kinase; mPTP, mitochondrial permeability transition pore; SNO, *S*-nitrosothiol; STAT3, signal transducer and activator of transcription 3; TKs, receptor tyrosine kinases.

Figure 3 illustrates the hypothetical pathways in the heart that are coupled with OT and the OTR.

The effects of OT that result in decreases in heart rate and inotropy, and vasodilation are mediated through the ANP-cGMP and NO-cGMP pathways (Gutkowska and Jankowski, 2011). Our study was performed using H9c2 cells initially isolated from ventricular muscle of the BDIX rat (Kimes and Brandt, 1976). However, H9c2 cells do not display pacemaker potentials and the ability to contract and have no proliferative capacity unlike true CMC. Despite these limitations, this cell line expresses CMC signaling pathways (Branco et al., 2015) and serves as an established *in vitro* model for the study of CMC in cardiac pathologies, including cardiac hypertrophy (Watkins et al., 2011). Studies confirm the characteristics of cardiac-like H9c2 cells in terms of their similarity to adult CMC and rates of mitochondrial respiration as well as relevance and vulnerability under conditions of IR (Botker et al., 2018). Using H9c2 cells exposed to simulated IR, we have investigated the role of OT on cell viability and resistance to apoptosis (Gonzalez-Reyes et al., 2015). Cell viability was preserved by activation of OTRs, whereas in cells expressing low OTR levels from siRNA-mediated knockdown, OT increased cell death under conditions of IR. Using this cell model, we recently demonstrated that OT treatment reduced cell respiration *via* specific OTRs (unpublished data). This observation is consistent with the findings reported by Boeck et al. (2018), who investigated oxygen consumption by high-resolution respirometry in peripheral blood mononuclear cells from mothers with a history of childhood maltreatment. The patients with high plasma OT levels showed reduced levels of oxygen consumption, whereas high cortisol was associated with enhanced respiration. The authors of this study suggested that high OT levels protected against increases in mitochondrial oxygen consumption, ROS production, and release of proinflammatory cytokines seen with stress and hypoxia. Treatment of infarcted rat hearts with OT resulted in favorable changes, including normalization of ANP mRNA expression in the LV scar area and increased eNOS synthesis. Treatment with OT also had pro-proliferative, anti-apoptotic, and anti-fibrotic effects (Jankowski et al., 2010b). In addition, we have demonstrated a robust effect of OT on glucose uptake in CMC *via* phosphoinositide-3-kinase (PI3K) and independent of insulin receptor signaling. This effect of OT was further increased in CMC with an uncoupler (2,4-dinitrophenol) of mitochondrial oxidative phosphorylation (Florian et al., 2010). The OTR is a GPCR, an integral membrane protein of the Gq/11α class and signaling activates phospholipase C-β (PLC-β) producing increases in intracellular levels of inositol-3-phosphate (IP3) and diacylglycerol (DAG) generation upon binding of OT. As a result, Ca^2+^ entry from the extracellular space and release from the sarcoplasmic reticulum are stimulated resulting in dissipation of the mitochondrial electrochemical gradient (Gravina et al., 2011). Activation of protein kinase C (PKC) by DAG signals pro-survival ERK and downstream targets, and intracellular Ca^2+^ mobilization stimulates the release of ANP from CMC (Schiebinger, 1989). Cardioprotection via phosphoinositide-3-kinases (PI3K)/Akt and NO has been reported with OT (Kobayashi et al., 2009; Florian et al., 2010; Polshekan et al., 2016). Moreover, in a rabbit model of MI, activation of STAT3 and ERK, key molecules that mediate survival signaling, was upregulated in ischemic areas of hearts treated with OT but not in control hearts (Kobayashi et al., 2009). Increased production of NO is beneficial on vascular and myocardial function and is a key molecule in angiogenesis involving vascular endothelial growth factor (VEGF) signaling through PI3K pathway (Papapetropoulos et al., 1997). The calcium-calmodulin kinase kinase and AMP-activated protein kinase (AMPK) pathways are also activated in the presence of OT in the CMC (Florian et al., 2010). Activation of the AMPK pathway is recognized as cardioprotective by reducing the extent of both apoptosis and cell damage after IR (Gelinas et al., 2018).

Signalosomes formed within the CMC represent a mechanism for increasing the specificity and efficiency of signal transduction pathways involved in protection (Penna et al., 2006; Negro et al., 2008). Assembled in caveolae (Cav), these vesicular and multimolecular signaling complexes target the outer mitochondrial membrane and disrupt the activity of mKATP channels, increasing the formation of ROS. This leads to activation of PKC epsilon and inhibition of mPTP in the mitochondria and decreasing myocardial injury (Pagliaro et al., 2018). In this regard, signalosomes by acting *via* a G-protein-coupled receptor are known to induce cardioprotection under both pre- and postconditioning conditions (Quinlan et al., 2008). It has been demonstrated that OTRs located inside and outside microdomains of caveolae initiate different signaling pathways; and depending on their localization, the OTR can transactivate EGFR and activate ERK1/2 using different signaling intermediates (Guzzi et al., 2002; Rimoldi et al., 2003). We have evidence that OT treatment stimulates the formation of signalosomes in CMC. In CMC, eNOS localizes to Cav-3, allowing eNOS activation by cell surface receptors and cellular surface NO release for intercellular signaling (Feron and Balligand, 2006). Using H9c2 cardiomyoblasts, we have demonstrated that IR increased Cav-3 expression with significant interaction with OT treatment. Further, immunofluorescence microscopy demonstrated that Cav-3 translocation from the cell surface to the cell perinuclear region occurs where mitochondria were concentrated (Gonzalez-Reyes et al., 2015). Co-localization of phosphorylated Akt with mitochondrial proteins and with the phosphorylated (Ser1177) form of eNOS substantiates the involvement of OT in signalosome formation (Gonzalez-Reyes et al., 2015). This effect is inhibited by KT-58235, an inhibitor of PKG (Garlid et al., 2009). Inhibition by this mechanism also blocks the beneficial effects of OT on CMC viability (Gonzalez-Reyes et al., 2015). The above-described mechanisms indicate pleiotropic molecular mechanisms involved in cardioprotection.

## Oxytocin in Pathways of Cardioprotection

In effect, the high intracellular concentrations of Ca^2+^ resulting from re-establishment of ionic homeostasis and the production of ROS trigger the opening of the mPTP, a large conductance pore that forms on the mitochondrial membrane. Sustained opening of the mPTP during reperfusion collapses the mitochondrial membrane potential and releases mitochondrial proteins into the cytosol, which in turn activates intracellular pathways that lead to cell death by apoptosis and necrosis (Bernardi and Di Lisa, 2015). During ischemia, the acidosis derived from reduced oxygen delivery prevents cell death by inhibiting the formation of the mPTP, and it is believed that CMC viability and infarct size can be dramatically improved by conditioning strategies (Davidson et al., 2019). Approximately 50% of the infarct size from cell death can be prevented by conditioning strategies. For these purposes, the preventative role of OT treatment on IR injury warrants further investigation. We and others have provided evidence that OTR-mediated signaling involves ANP-cGMP and NO-cGMP pathways in the cardiovascular system, which are associated with regulation of cardiac function and vascular tone (Gutkowska and Jankowski, 2012; Gutkowska et al., 2014). Stimulation of these pathways with OT reduces the force and rate of contraction (Mukaddam-Daher et al., 2001), promotes vasodilation (Jankowski et al., 2000), and provides protection against MI in rats preconditioned with OT (Jankowski et al., 2010b; Ondrejcakova et al., 2012). However, Polshekan et al. (2016) have demonstrated by using the isolated perfused rat heart model that OT exhibits anti-ischemic effects when acutely administered at the onset of reperfusion. Similarly, we also demonstrated this protective effect using H9c2 cells exposed to experimental IR in the presence of OT with optical effects of OT observed at the onset of reperfusion (Gonzalez-Reyes et al., 2015). Therefore, it suggested that cardioprotection from OT has predominantly postconditioning effects by activation of the Reperfusion injury salvage kinase (RISK) pathway, which consists of a combination of two parallel cascades. These include the PI3K-Akt and MEK1-ERK1/2 pathways and their purported signal convergence through activation of cGMP-dependent protein kinase (PKG) and PKCε, preventing prolonged mPTP opening (Rossello and Yellon, 2018). More recently, Polshekan et al. (2019) reported inhibition of experimental infarct after administration of OT in isolated perfused male rat hearts at the early phase of reperfusion through activation of the Janus kinase (JAK)/signal transducer and signal transducer and activator of transcription protein 3 (STAT3) signaling pathway and NO release. This pathway, termed survivor activating factor enhancement (SAFE), is linked with the activation during preconditioning (Rossello and Yellon, 2018). In our study, we demonstrated the action of OT occurring with the third signaling cascade based on the PKG and involving NO and/or ANP release (Jankowski et al., 2010b), which has been also proposed to mediate cardioprotection (Cohen and Downey, 2007). The degree of crosstalk between these protective pathways induced by OT is presently unclear, but it is possible that they ultimately protect mitochondrial function (Heusch et al., 2008). Pharmacotherapy directed to these pathways may represent an attractive alternative to ischemic conditioning (Gross and Gross, 2006). The negative inotropic effect triggered by OT preconditioning was linked to myocardial protection against infarction (Ondrejcakova et al., 2009). On a molecular level, OT treatment can modify numerous paracrine factors released by CMC and other cardiac cells under ischemic conditions, leading to functional benefits on the heart (Noiseux et al., 2012). In fact, OT preconditioning increases the synthesis of HSP27, HSP32, HSP70, and VEGF in heart collectively favoring angiogenic, anti-apoptotic, and anti-remodeling mechanisms (Noiseux et al., 2012). Moghimian et al. (2014) observed a four to fivefold increase in cardiac expression of Hsp27 from stress induction and intracerebroventricular infusion of OT in the isolated perfused rat heart. As reviewed by Penna et al. (2018), HSP27 and HSP70 are molecular chaperones produced by the CMC under stress. These chaperones, in particular HSP70, are translocated into mitochondria where their proteins regulate cell differentiation and survival (Penna et al., 2018). Depending on the inflammatory response, HSP70 either initiates proinflammatory signals or modulates specific anti-inflammatory pathways, reducing apoptotis (Joly et al., 2010). Both HSP27 and HSP70 are elevated in the skeletal and cardiac muscles after exercise training (Kruger et al., 1985; Penna et al., 2018), a potent stimulus of OT system in these organs (Gutkowska et al., 2007). With exercise training, expression of these stress proteins occurs simultaneously with the synthesis of expression antioxidants, providing homeostatic adaptations to training (Penna et al., 2018). It is especially important that both HSP70 and HSP27 induce cardioprotection against irreversible injury associated with IR and that HSP27 is induced in the rat heart by intracerebroventricular injection of OT (Moghimian et al., 2014). In summary, there are numerous mediators playing a role in OT-mediated heart protection.

## Effects of Oxytocin on Mitochondrial Function

Do the aforementioned mechanisms explain the effects of OT on mitochondria? Mitochondrial dysfunction plays a critical role in many cardiac pathologies including IR, resulting in ventricular systolic dysfunction and compensatory cardiac hypertrophy (Pagliaro et al., 2018). Disturbances in both function and structure of mitochondria can lead to irreversible injury during IR. During reperfusion, rapid removal of intracellular protons coupled with a sudden and dramatic increase in Ca^2+^ entry into the CMC leading to subsequent Ca^2+^ overload in the mitochondria results in cell damage, decreased cell viability, and decreased contractile function (Botker et al., 2018). It is proposed that cardioprotective signaling pathways target mitochondria, and as a consequence, various mitochondrial proteins are post-transitionally modified with both pre- and postconditioning (Heusch, 2017; Pagliaro et al., 2018). In this context, several studies postulate the presence of different beneficial signal transduction pathways that converge on mitochondria, thus reserving function relating to mKATP channels, the mPTP function, connexin 43, and the large conductance calcium-activated potassium channel (mitoBKCa) (Botker et al., 2018; Pagliaro et al., 2018; Penna et al., 2020a).

The OTR with pAkt is translocated from the cell surface to central structures when cells are treated with OT (Klein et al., 2011), suggesting the importance of intercellular communication mechanisms and molecular trafficking in OT signal transduction. Correspondingly, IR studies using isolated rat hearts suggest that the beneficial OT effects are inhibited by the mitochondrial ATP-dependent potassium channel (Alizadeh et al., 2010). Mitochondria are essential in regulating survival pathways and cell death; and because of this role, the protective effect of OT is likely associated with reduced injury to the mitochondria. Therefore, we believe that that OT mediates protection of mitochondrial function in conditions of hypoxia and oxidative stress by G-protein-coupled receptor/ANP/NO signaling and stimulating specific paracrine factors that will approve cell proliferation and prevent cell death (Gutkowska et al., 2000; Florian et al., 2010; Jankowski et al., 2010b; Noiseux et al., 2012; Gonzalez-Reyes et al., 2015).

While direct evidence connecting OT to mitochondrial function is limited, some recent data suggest that there is a direct link. The modulation of electron transport is recognized as a mechanism associated with cardiac mitochondrial protection, which reduces myocardial injury during ischemia and early reperfusion (Pagliaro et al., 2018). Consistent with this effect, Gravina et al. (2011) demonstrated that OT depolarizes the mitochondrial membrane potential in isolated myometrial cells. By depolarizing the membrane potential of these cells, activity of the mitochondrial membrane ATP synthase was increased from the initial rise in the intracellular concentration of Ca^2+^ (Gravina et al., 2011). In order to induce cardioprotection by postconditioning in isolated perfused rat hearts, signaling through mKATP activation and a redox-sensitive mechanism is required (Penna et al., 2006; Pagliaro et al., 2018). Correspondingly, Alizadeh et al. (2010) demonstrated that the beneficial effects of OT on myocardial injury in the ischemic reperfused rat heart were reduced by 5-hydroxydecanoate, an inhibitor of the mitoKATP channel, and atractyloside, an opener of the mPTP (Alizadeh et al., 2012). As recently reported, cardiac mitochondria express mitoBKCa and cGMP-dependent protein kinase Iα (cGKIα) (Frankenreiter et al., 2018). Upon activation via NO or ANP pathways, phosphorylation of cGKI leads to increased opening of the mitoBKCa channels. This provides evidence to suggest that this mechanism is linked with the capacity of mitochondria to tolerate Ca^2+^ loading and the closure of the mPTP and to induce cardioprotection (Hoffmann and Spengler, 2018).

We found that treatment of the D3 stem cell line with OT resulted in an increase in connexin 43 protein (Gassanov et al., 2008), and recent observations suggest a beneficial role of mitochondrial connexin 43 in postconditioning-induced ROS signaling, although the precise function is not clear (Pagliaro et al., 2018). Further evidence linking OT to mitochondrial function stems from our previous study showing that OT treatment is protective against cell death and mitochondrial dysfunction, expressed as succinate dehydrogenase activity (MTT assay), and provided protection against ROS in H9c2 cells subjected to simulated IR (Gonzalez-Reyes et al., 2015). We have also reported the paradoxical effect of OT on ROS production during ischemia; OT either stimulates the production of moderate levels of ROS or inhibits the production of ROS (Gonzalez-Reyes et al., 2015). In fact, some ROS-induced signals play important physiological roles as intracellular mediators of vasodilatation, cell growth, and angiogenesis (Tullio et al., 2013) with earlier evidence indicating the involvement of OT in these effects (Gutkowska and Jankowski, 2012). Indeed, moderate levels of ROS function as signaling molecules involved in protection of CMC. Under these conditions, ROS activate Pi3K/Akt within and outside the mitochondria (Penna et al., 2020a). ERK1/2, MAPK p38, and/or JAK/STAT pro-survival signaling cascades are also activated with moderate levels of ROS (Davidson et al., 2019). We recently observed that OT preconditioning of cardiac cells decreased oxidative and apoptotic activity in doxorubicin (Dox)-induced toxicity in H9c2 cells (unpublished data). With the use of formazan production for the measurement of cell viability and metabolic activity, the effect of OT was investigated in conditions of cytotoxicity induced by Dox in H9c2 cells as well as in the absence of Dox treatment. Preconditioning with OT (at the concentrations of 62.5 and 1,000 nM) for a period of only 30 min prior to the administration of Dox improved viability of H9c2 cells. Dox is commonly used for chemotherapy, but its use is limited due to myocardial damage and disrupted mitochondrial function, leading to excessive production of superoxide radicals (Wallace, 2003). Interestingly, myocardial injury due to Dox toxicity is associated with activation of the NLRP3 inflammasome (Marchetti et al., 2015), which suggests that Dox-induced cardiotoxicity can be reduced with an NLRP3 inflammasome inhibition. The effects of OT treatment on Akt phosphorylation and translocation into mitochondria in CMC are consistent with the benefits of reduced oxidative stress and toxicity from Dox (Fan et al., 2008; Gonzalez-Reyes et al., 2015). A recent report indicated that the improvement in cardiac function following OT treatment was associated with decreased oxidative stress, apoptosis, and inflammation in a rat model of doxorubicin-induced cardiomyopathy (Taskiran et al., 2019). Similarly, Kaneko et al. (2016) reported that pretreatment with OT increased cell viability and decreased mitochondrial damage in rat neural cells exposed to oxygen-glucose deprivation IR. Xu et al. (2017) reported that a beneficial effect of OT is related to increase of superoxide dismutase in skin cells, the antioxidant enzyme, which catalyzes the conversion of superoxide to the harmless components of oxygen. Further studies are required to better clarify molecular mechanism OT involvement in mitochondrial survival and function.

## Effects of Oxytocin on Cardiomyocyte Hypertrophy

Hypertrophic growth of the heart predisposes to multiple cardiovascular and cerebrovascular events. This abnormal growth of the CMC in response to hemodynamic stress is mediated by growth factors and cytokines acting via specific G protein isoforms, low-molecular-weight GTPases, mitogen-activated protein kinases, and PKC. We have reported that chronic OT treatment in various diseased animal models, including MI (Jankowski et al., 2010b), ovariectomy (Jankowski et al., 2010b), and diabetic cardiomyopathy (Plante et al., 2015), significantly reduced the development of CMC hypertrophy. The significant increase in CMC volume resulting from infarction in rats was reduced by OT treatment, and we hypothesized that this effect could be mediated by the accumulation of ANP in CMC (Menaouar et al., 2014). Recently, Jovanovic et al. (2019) reported that OT treatment decreased the heart/body weight ratio and prevented the hypertrophy of CMC in the wall of the LV of rats subjected to social isolation. The authors suggested that the beneficial effects of OT were associated with enhanced expression of β_3_-adrenoceptors and muscarinic M_2_ receptors in the heart. The mechanism of this anti-hypertrophic action is unknown because stimulation of muscarinic M_2_ receptors is known to stimulate (Kim H.Y. et al., 2013) ANP release, whereas activation of β_3_-adrenoceptors prevents ANP release from the heart (Luchner and Schunkert, 2004).

One of the major pathways in the pathological hypertrophic response to increased growth factor expression is the calcineurin-NFAT (nuclear factor of activated T cells) pathway. We have reported that OT regulates this pathway in CMC hypertrophy induced by ET-1 and Ang-II. In physiological concentrations (∼10 nM), OT prevented hypertrophy in newborn and adult rat CMC by several mediators, including PI3K/ERK1/2/ANP-cGMP/NFAT signaling (Menaouar et al., 2014). In this study, we demonstrated that OT stimulated the production of ANP in CMC independently of cell hypertrophy. Secondly, OT supplementation to CMC cultures stimulated the production of cGMP in cells by mechanisms involving soluble and particulate guanylyl cyclases. In effect, OT-preconditioned CMC were resistant to the hypertrophic stimulus of ET-1 (Menaouar et al., 2014). Signaling *via* particulate guanylate cyclase receptors (NPR-A/NPR-B) and cGMP by NP production is a well-known mechanism that inhibits pathological hypertrophy (Oliver et al., 1997). Our work is consistent with the observations that stimulation of cGMP-PKG I blocks pathological hypertrophy by inhibiting the NFAT pathway (Fiedler et al., 2002). This mechanism was ineffective in insulin-induced hypertrophy of CMC. These observations indicate that OT specifically counteracts pathological heart hypertrophy.

## Conclusion

In summary, OT is produced in the cardiovascular system and is critical in the regulation of cardiac and vascular function during early development and adulthood. The major actions of OT include regulation of chronotropy and inotropy of the heart as well as vascular tone of cardiac resistance vessels. OT is also involved in blood pressure and body volume regulation via the cardiac–renal axis and the release of ANP and NO. In addition to cardiovascular regulation and protection, OT exerts robust anti-oxidative and anti-inflammatory effects in CMC. In cells, OT targets mitochondria, promoting glucose uptake and reducing the effects of ROS upon reperfusion.

Animal studies indicate that OT is not only a cardiovascular protective peptide but also critical for cardiovascular homeostasis and in the reduction in the severity of cardiovascular pathologies. The significance of OT in cardioprotective signaling warrants further study.

## Author Contributions

MJ, TB, and JG equally contributed to the organization and writing the manuscript. All authors contributed to the article and approved the submitted version.

## Conflict of Interest

The authors declare that the research was conducted in the absence of any commercial or financial relationships that could be construed as a potential conflict of interest.
